# Functional organization of the midbrain periaqueductal gray for regulating aversive memory formation

**DOI:** 10.1186/s13041-021-00844-0

**Published:** 2021-09-08

**Authors:** Li-Feng Yeh, Takaaki Ozawa, Joshua P. Johansen

**Affiliations:** 1 RIKEN Center for Brain Science, Laboratory for the Neural Circuitry of Learning and Memory, 2-1 Hirosawa, Wako-shi, Saitama 351-0198 Japan; 2 Department of Life Sciences, Graduate School of Arts and Sciences, University of Tokyo, 3-8-1 Komaba, Meguro-ku, Tokyo, 153-8902, Japan; 3 Institute for Protein Research, Osaka University, 3-2 Yamadaoka, Suita-shi, Osaka, 565-0871 Japan

**Keywords:** Fear conditioning, Brainstem, Thalamus, Periaqueductal gray

## Abstract

Innately aversive experiences produce rapid defensive responses and powerful emotional memories. The midbrain periaqueductal gray (PAG) drives defensive behaviors through projections to brainstem motor control centers, but the PAG has also been implicated in aversive learning, receives information from aversive-signaling sensory systems and sends ascending projections to the thalamus as well as other forebrain structures which could control learning and memory. Here we sought to identify PAG subregions and cell types which instruct memory formation in response to aversive events. We found that optogenetic inhibition of neurons in the dorsolateral subregion of the PAG (dlPAG), but not the ventrolateral PAG (vlPAG), during an aversive event reduced memory formation. Furthermore, inhibition of a specific population of thalamus projecting dlPAG neurons projecting to the anterior paraventricular thalamus (aPVT) reduced aversive learning, but had no effect on the expression of previously learned defensive behaviors. By contrast, inactivation of dlPAG neurons which project to the posterior PVT (pPVT) or centromedial intralaminar thalamic nucleus (CM) had no effect on learning. These results reveal specific subregions and cell types within PAG responsible for its learning related functions.

**Supplementary Information:**

The online version contains supplementary material available at 10.1186/s13041-021-00844-0.

## Introduction

Innately aversive experiences produce immediate defensive behaviors and engage instructive circuits to trigger long lasting memories. The midbrain periaqueductal gray (PAG) coordinates defensive behavioral responses to innately and learned aversive events through afferent inputs from aversive sensory systems and forebrain regions like the amygdala and hypothalamus important for processing information related to learned and social threats [1–4]. For example, various forms of escape behavior are encoded in dorsolateral PAG (dlPAG) neurons [5–8] and stimulation of dlPAG produces escape behaviors [7, 9, 10]. Projections from hypothalamus to the dlPAG regulate escape responses from predators [11] while projections from the amygdala to PAG engage learned behavioral freezing and autonomic responses to sensory cues which have previously been associated with noxious stimuli [12]. The PAG then controls defensive motor and autonomic responses through projections to brainstem regions connected to the spinal cord and autonomic nervous system [10]. As suggested by the function of different afferent inputs from amygdala and hypothalamus to PAG, distinct PAG subregions regulate distinct types of defensive responses [1, 13]. In response to sensory cues that predict danger, the vlPAG engages passive defensive responses including freezing, bradycardia and analgesia, while the dlPAG controls active escape responses to threats.

While the role of the PAG in producing innate and learned defensive responses is well established, some studies suggest that the PAG also participates in instructing learning in response to aversive stimuli during auditory fear conditioning [14–16]. During fear conditioning, animals learn that an innocuous auditory stimulus (conditioned stimulus or CS) predicts the occurrence of an aversive outcome (unconditioned stimulus or US, typically footshock) [17, 18]. Following learning, animals exhibit a variety of behavioral and autonomic responses upon presentation of the previously neutral auditory cue including behavioral freezing. Prior studies reported that PAG neurons receive synaptic input directly from the spinal cord dorsal horn and are shock responsive [14, 19–21]. Furthermore, inactivation of PAG neurons reduces shock responsiveness in neuron in the lateral nucleus of the amygdala (LA) [14] where shock evoked depolarization is thought to produce synaptic plasticity underlying fear learning and memory [22–26]. Finally, stimulation of dlPAG, but not vlPAG, produces fear learning which is dependent on neural activity in the lateral amygdala [2, 15]. Although the PAG does not project directly to the LA,  it has been hypothesized that PAG projections to the thalamus convey aversive instructive information to the LA  and other forebrain structures to produce learning [27]. Together, this work suggests that the dlPAG is important for aversive associative learning, possibly through a population of PAG neurons which project to the thalamus. However, the PAG subregions and cell types which are necessary for producing aversive learning have not been identified. Furthermore, whether, and if so which, PAG projections to the thalamus are important in memory formation is not known.

## Results

**Fig. 1:**
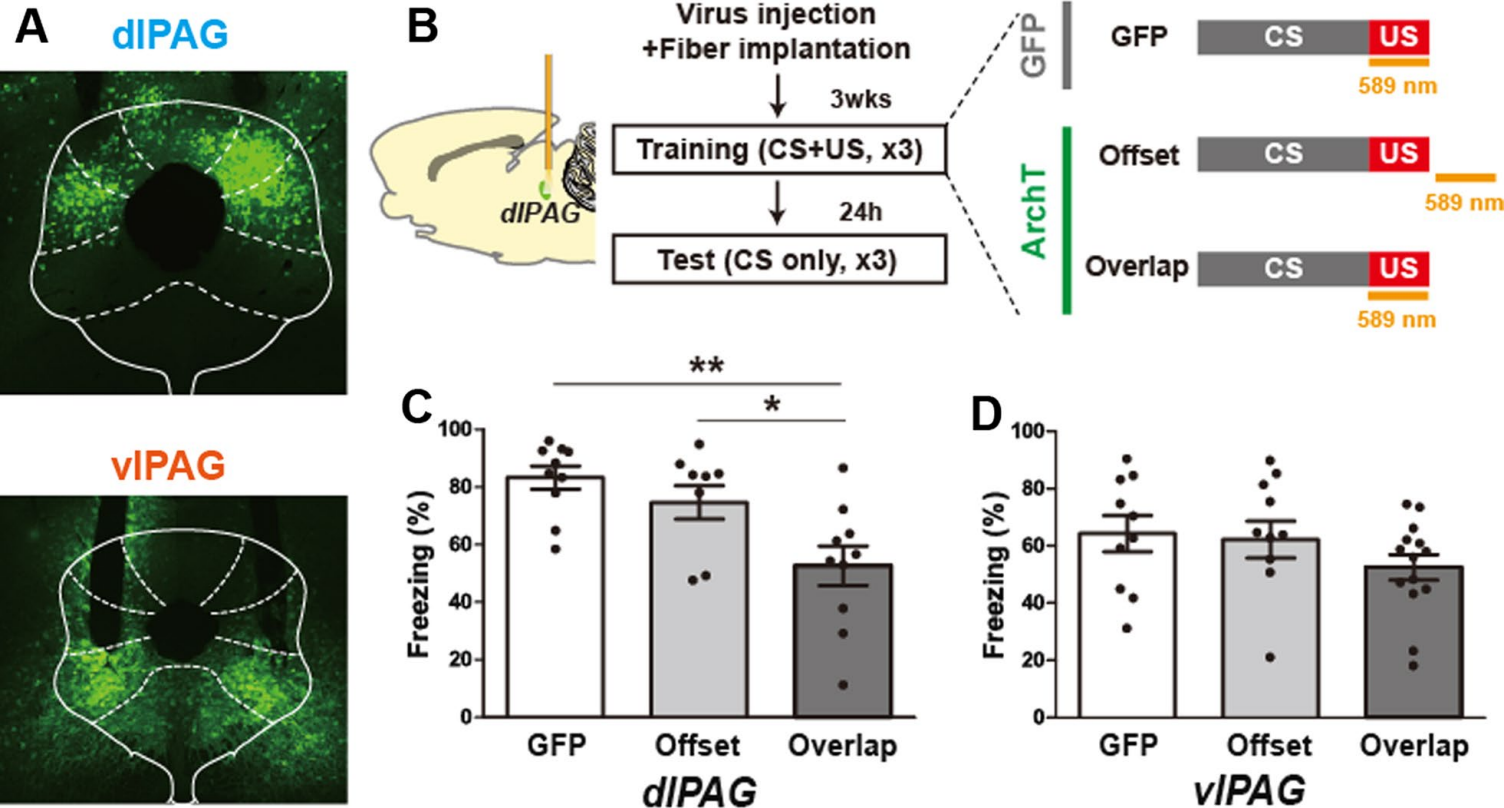
**A** Example picture of virus expression in dorsolateral (dl, top) and ventrolateral (vl, bottom) PAG. **B** Experimental design and optogenetic manipulation during fear conditioning as well as experimental groups. **C** Fear memory formation is reduced when dl/lPAG neurons are inhibited during the shock period of fear conditioning. Auditory CS-evoked behavioral freezing responses during 'Test' period following learning. GFP (n = 10), ‘Offset’ (n = 9) and ‘Overlap’ (n = 10) groups. A one way ANOVA revealed significant main effect of optogenetic manipulation [F(2, 26) = 7.93, p = 0.002] and post-hoc test showed that averaged CS-evoked freezing during all three CSs in the ‘Overlap’ group is significantly lower than that of the ‘GFP’ and ‘Offset’ control groups (*p < 0.05, **p < 0.01). **D** Optogenetic inhibition of vlPAG during the shock period of fear conditioning had no affect on the acquisition of fear memory formation [F(2, 32) = 1.35, p = 0.27]. GFP (n = 14), ‘Offset’ (n = 11) and ‘Overlap’ (n = 10) groups

To determine which PAG subregions are important in fear learning, we used an optogenetic strategy to inhibit neurons in the vlPAG or dlPAG during the aversive shock period of auditory fear conditioning and examined the effects of these manipulations on auditory cue-evoked freezing responses, a behavioral measure of memory, 24 h later. Specifically, we injected adeno-associated virus expressing the hyperpolarizing archaerhodopsin [28, 29] or control vector (AAV5-CAG-ArchT-GFP or AAV5-CAG-GFP) into the vlPAG or dlPAG followed by implantation of fiber optic cables above the injection site (Fig. 1A, B, Additional file 1: S1A). During the fear conditioning/training phase, we then attached the fiber optic cables to a laser and shone orange light to inhibit vlPAG or dlPAG neuronal activity during (‘Overlap’ group and a ‘GFP’ control group) or after (Offset group) the shock period of fear conditioning and examined the effects of these manipulations 24 h later at the memory test point (Fig. 1B). We found that inactivation of dlPAG, but not vlPAG, neurons reduced the acquisition of aversive memories in animals that received laser inhibition during the shock period of fear conditioning (‘Overlap’ group) relative to control animals (Fig. 1C, D).

**Fig. 2:**
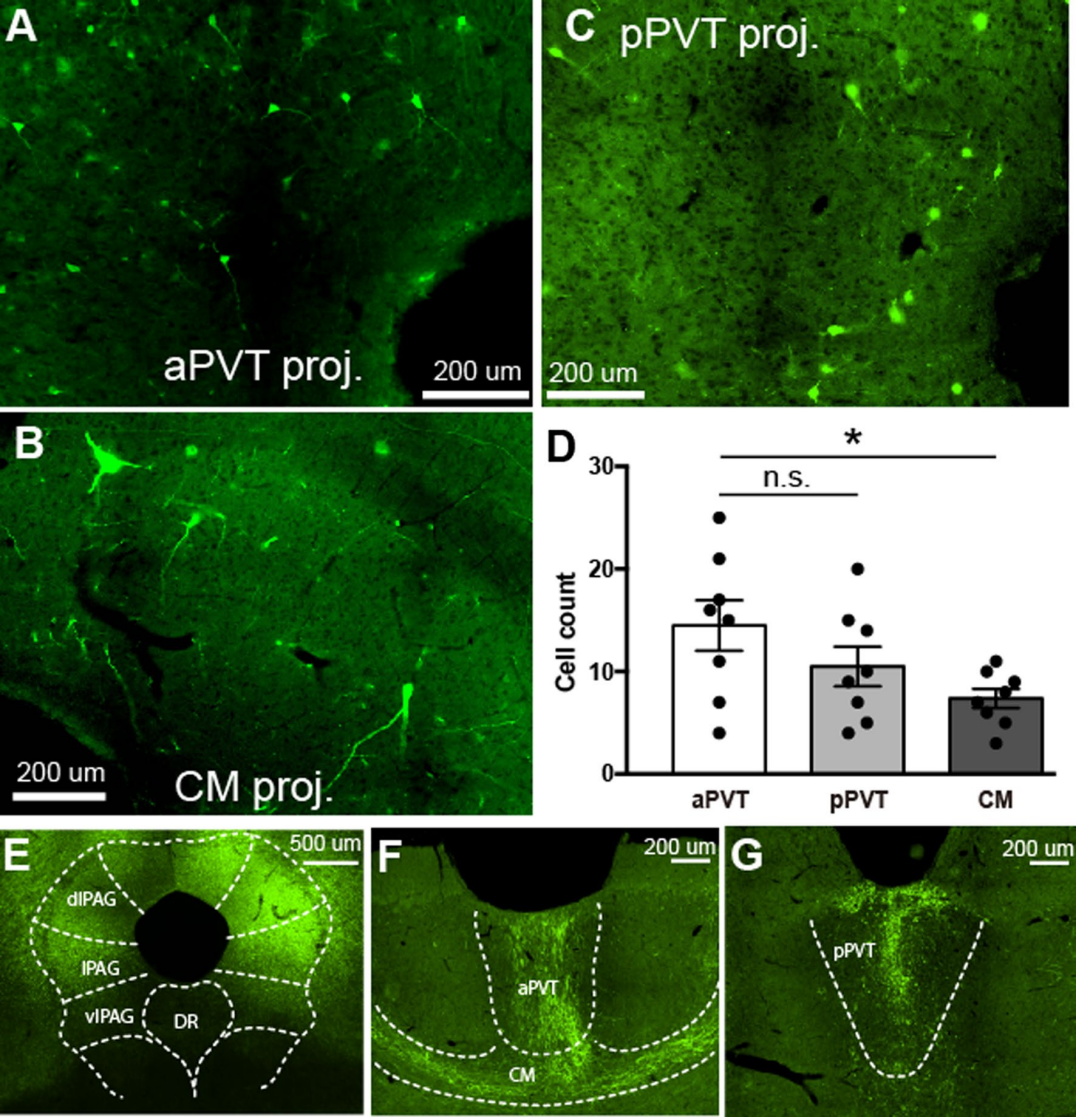
**A**–**C** Example of retrogradely labeled dlPAG neurons projecting to the aPVT (**A**), CM (**B**) and pPVT (**C**). **D**, quantification of the number of dlPAG neurons labeled after injection of retrograde virus into aPVT (n = 8), pPVT (n = 8) and CM (n = 8). A one way ANOVA revealed a significant main effect of group [F(2, 21) = 3.57, p = 0.046] and post-hoc tests revealed no significant difference between the number of aPVT and pPVT projecting cells, but a significantly smaller number of CM compared with aPVT projecting dlPAG neurons (*p < 0.05). **E–G** Efferent axonal projections of dlPAG neurons to midline thalamic nuclei. **E** EYFP expression following virus injection into dlPAG. EYFP labeled axons in aPVT, CM (**F**) and pPVT (**G**) following virus injection into dlPAG

Having identified a specific dlPAG subregion important for aversive learning, we next examined whether specific populations of dlPAG neurons which send axonal projection to thalamic regions are responsible for producing memory formation. We selected three midline thalamic nuclei, the anterior and posterior portions of the paraventricular nucleus (aPVT and pPVT) and the intralaminar centromedial nucleus (CM), as a previous study suggested that these regions receive input from the PAG [30] and also project to the LA [31–33]. The PAG projects to other midline thalamic nuclei including the centrolateral intralaminar nucleus, but these were not considered because they do not project to the LA. To test the anatomical connectivity between dlPAG and the aPVT, pPVT and CM using viral tracing approaches, we first injected a retrograde rabies virus [34] expressing archaerhodopsin-EYFP (eArchT3.0-EYFP) [35] into either aPVT, pPVT or CM and examined cell body labeling in the dlPAG. We found that injections into all of these regions produced moderate cell labeling in the dlPAG (Fig. 2A–C). While there was no significant difference in the number of EYFP + aPVT projecting compared with the number of pPVT projecting neurons, there was a significantly smaller number of cells projecting to CM compared with aPVT (Fig. 2D). We then injected an anterograde viral tracer (adeno-associated virus expressing EYFP) into dlPAG and identified substantial axonal labeling in all three thalamic regions (Fig. 2E–G). Thus, dlPAG neurons project substantially to all three midline thalamic nuclei.

**Fig. 3:**
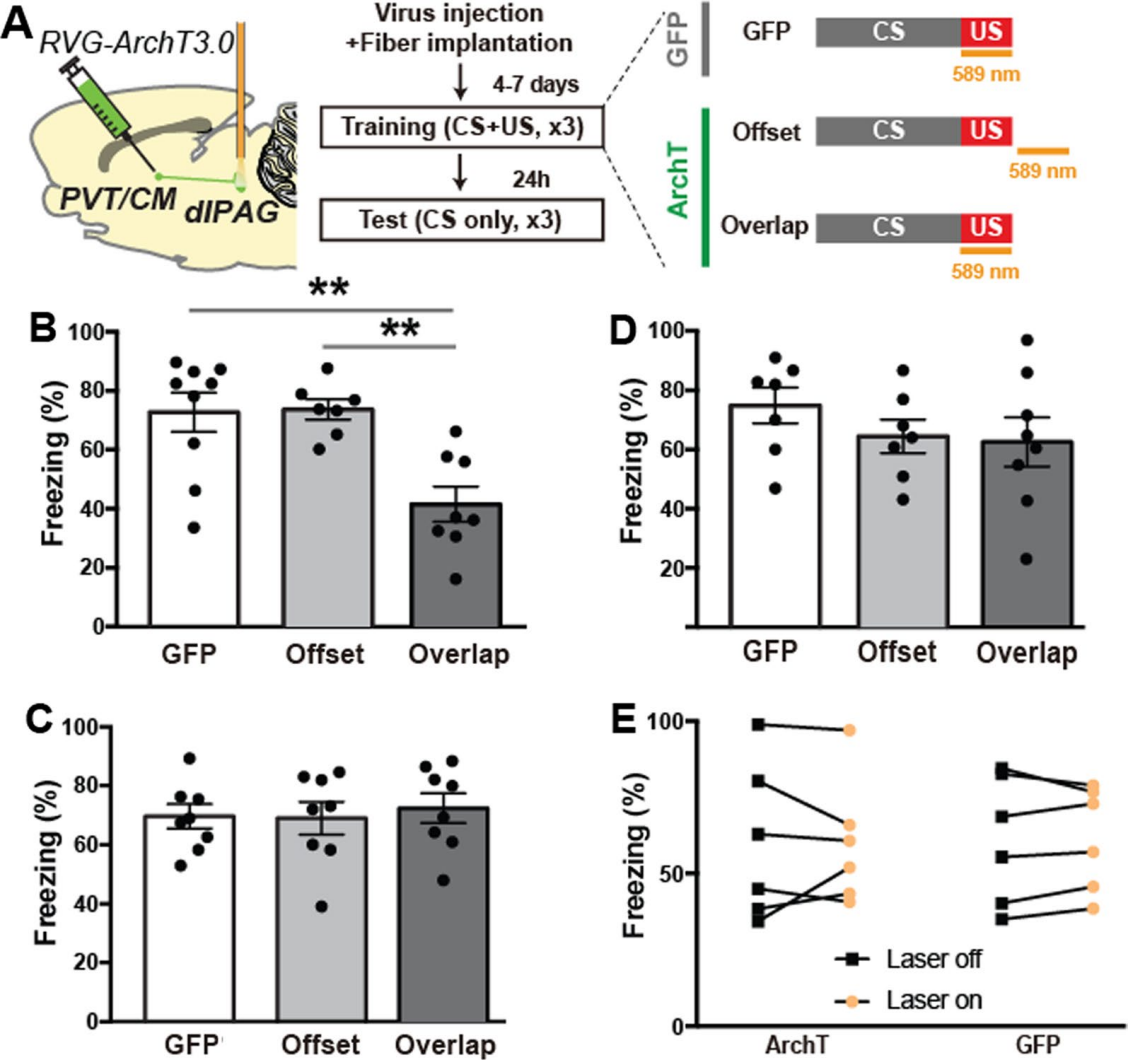
**A** Experimental protocol for inactivation of PAG projection neurons during fear learning. **B** Inactivation of aPVT projecting dlPAG neurons reduced fear learning. A one way ANOVA revealed significant main effect of optogenetic manipulation [F(2, 21) = 9.87, p = 0.001] and post-hoc test showed that averaged CS-evoked freezing during the Test time-point in the ‘Overlap’ group (n = 8) was significantly lower than that of the ‘GFP’ (n = 9) and ‘Offset’ (n = 7) control groups (**p < 0.01). **C**, **D** Inactivation of pPVT (**C**, [F(2, 21) = 0.13, p = 0.875]; n = 8 for all groups) and CM (**D**, [F(2, 19) = 0.92, p = 0.417]; Overlap n = 8, Offset n = 7, GFP n = 7) projecting dlPAG neurons had no effect on fear learning. **E** Inactivation of aPVT projecting dlPAG neurons during auditory CS presentation following learning had no effect on the expression of previously learned fear responses. 2-way ANOVA interaction [F(1, 21) = 0.0009, p = 0.9752], n = 6 for both groups

We next examined whether activity in dlPAG neuron populations which project to one of these thalamic regions is necessary to produce aversive learning in response to shock. To test this we used an optogenetic approach to inactivate dlPAG neurons projecting to aPVT, pPVT or CM by injecting a retrograde rabies virus expressing eArchT3.0-EGFP into these thalamic regions in individual experiments, followed by implantation of fiber optic cables into the dlPAG (Fig. 3A). We then inactivated the thalamic projecting dlPAG neurons during the shock US period of auditory fear conditioning in separate experiments and compared this to animals which had received EGFP expressing virus ('GFP') or eArchT3.0-EGFP with the ‘Offset’ treatment (Fig. 3A). We found that inactivation of aPVT, but not pPVT or CM, projecting dlPAG neurons reduced fear learning (Fig. 3B–D, Additional file 1: S1B–D). Furthermore, the number dlPAG neurons which were retrogradely labeled was significantly negatively correlated with the amount of freezing animals expressed 24 h after learning (i.e. higher expression of eArchT3.0 in dlPAG was correlated with reduced memory formation) in the aPVT (r = − 0.64, p = 0.048), but not the CM (r = 0.14, p = 0.38) or pPVT (r = − 0.02, p = 0.49), ‘overlap’ groups (Additional file 2: S2A–C). Importantly, inactivation of aPVT projecting dlPAG cells had no effect on the expression of previously learned freezing responses (Fig. 3E), demonstrating that this cell population does not participate in producing previously acquired learned freezing response. Next, to determine whether the aPVT projecting dlPAG cells are glutamatergic, we retrogradely tagged them (using the retrograde tracer Ctb647 injected into aPVT) and immunohistochemically labeled dlPAG cells with vGluT2, a marker of glutamatergic neurons. We found that aPVT projecting dlPAG neurons are almost exclusively glutamatergic (vGluT2 + , Additional file 2: S2D, E). Finally, to determine whether the aPVT projecting cells collateralize to CM or pPVT we injected (n = 3) a cocktail of two retrograde viruses, both expressing cre-recombinase (canine adenovirus, CAV-cre and retrograde traveling AAV, retroAAV-cre) followed by injection of a cre-dependent AAV virus expressing green fluorescent protein (AAV-flex-GFP) into the dlPAG and examined the expression of axon collaterals in other regions. No axons were detected in CM or pPVT, but axons were seen consistently in other regions such as the dorsomedial hypothalamus (DMH), cuneiform nucleus (CnF) and parabrachial nucleus (PB) (Additional file 2: S2F, G). Together, these results show that a population of glutamatergic dlPAG neurons which project to the aPVT are important for fear memory formation, but not expression of previously learned fear responses.

## Discussion

Together, these results demonstrate that during innately aversive experiences, activity in dlPAG, but not vlPAG, neurons is important for producing fear learning. Furthermore, these findings implicate a specific population of dlPAG glutamatergic neurons which project to the aPVT. While earlier studies reported that stimulation of dlPAG was sufficient to produce aversive learning [15, 16], the present findings demonstrate that activity in dlPAG cells during an aversive experience is necessary for aversive memory formation.

Our findings that dlPAG projections to aPVT support fear learning, while projections to pPVT do not, suggests that different afferent inputs can regulate distinct functions in these two PVT subregions. Previous experiments implicated the PVT in both aversive and reward processing [36–39]. Recent studies reported that distinct populations of aPVT cells projecting to different efferent targets facilitate aversive or reward learning and inhibit arousal while a class of genetically defined pPVT cells signal reward [40, 41]. Importantly, stimulation of PVT projections to the amygdala or ventral striatum produces place aversion [41]. While we found that aPVT projecting dlPAG neurons serve an aversive instructive function, it is unclear whether a dlPAG-aPVT-amygdala pathway is responsible for aversive learning. In fact, these aPVT projecting dlPAG cells also collateralize to other brain regions in the brainstem which could participate in aversive learning (Additional file 2: S2F, G). Further studies will be needed to determine whether  synaptic inputs of dlPAG neurons to aPVT (and specific aPVT cell types which project to the LA) participate in aversive learning.

Although the the role of distinct PAG sub-regions in eliciting different types of defensive responses has been extensively studied, how PAG circuits regulate learning is not as well understood. Opioid receptor activation in vlPAG is known to limit the strength and specificity of learning [42], suggesting that it could be part of a negative feedback circuit which inhibits aversive processing when aversive outcomes are anticipated and thereby regulates learning. Relatedly, recent studies found that vlPAG neurons respond to and encode the value of auditory CSs and this information could be used as a negative feedback signal that is activated by cues that predict danger [14, 43, 44]. Furthermore, inputs from the CeA to vlPAG are necessary for auditory CSs to activate a specific population of vlPAG neurons projecting to pain modulatory sites in the brainstem to control the adaptive strength of fear learning in response to different intensities of aversive experience [43]. Consequently, inhibition of these brainstem projecting vlPAG neurons or all vlPAG neurons enhances the strength of aversive associative learning [43, 45]. The CeA-vlPAG negative feedback pathway ultimately does this by inhibiting shock processing in various brain regions involved in fear learning, including in amygdala and dlPAG neurons, when the shock is predicted by a well-trained auditory CS. This limits depolarization of LA pyramidal neurons and thereby restrains learning [43]. These results along with prior reports that stimulation of PAG neurons is sufficient to produce aversive learning [15, 16] and the present findings showing that neural activity in a specific population of dlPAG neurons during the shock is necessary to produce learning support a revised model of PAG function in instructive signaling. According to this model, dlPAG provides instructive signals to forebrain centers in response to aversive events to produce learning while vlPAG neurons are activated by cues predicting noxious stimulation to restrain learning and set memory strength proportional to the intensity of the aversive experience.

The PAG (and many other brainstem regions) is a sensorimotor structure, but how sensory and motor information is encoded and used by different PAG subregions and cell types to both control behavior and initiate learning is not known. One possibility is that aversive sensory information is routed from PAG to the forebrain to inform the organism about innately aversive events in the external world to initiate emotional states and learning, while defensive motor information is encoded in PAG neurons projecting to brainstem motor structures. Alternatively, both sensory and motor information may be encoded in and conveyed to brainstem and forebrain structures. In this scenario, forebrain projecting dlPAG neurons may encode information about noxious stimuli and the escape responses these stimuli elicit. This could then be used by forebrain structures such as the amygdala to construct a representation of the external-sensory and internal-motor aspects of emotion inducing experiences which can drive learning. Answering this question is important as there is an old, but unresolved debate about whether emotional states represent sensory or motor aspects of emotion inducing experiences [46, 47]. One possibility is that dlPAG neurons projecting to brainstem motor regions initiate escape responses during aversive experiences (e.g. pain, visually threatening stimuli, etc.) while ascending dlPAG cells convey information about sensory and motor aspects of these experiences to produce emotional representations in forebrain structures which function to instruct learning as well as modulate and further coordinate ongoing behavioral responses. Answering this question may provide insights into how brainstem sensorimotor structures like the PAG support emotional state encoding in forebrain structures to regulate learning and behavior.

## Methods

### Subjects

Male Sprague–Dawley rats weighing 250–275 g were singly housed on a 12 h light/dark cycle and given food and water ad libitum. Experimental procedures were approved by the Animal Care and Use Committees of the RIKEN Brain Science Institute.

### Plasmids and viral vectors

AAV5-hSyn-EYFP, AAV5-CAG-ArchT-GFP and AAV5-CAG-GFP were produced and packaged by the University of North Carolina Vector Core. CAV2-cre was produced and packaged at Montpellier Vectorology. retroAAV2-Ubiq-mCRE virus as well as Rabies virus [34] (RV∆G)-eArchT3.0-EGFP and RV∆G-EGFP were produced and packaged in the Johansen laboratory.

### Stereotaxic cannula implantation and virus injection

For all surgeries, animals were injected intraperitoneally with a mixture of ketamine (100 mg/kg) and Xylazine (10 mg/kg) and supplemental doses were given as required. For behavioral experiments where the vlPAG and dl/lPAG cell bodies or specific thalamic projecting dlPAG cells were optogenetically manipulated, following anesthesia, animals were placed in a stereotaxic frame (Leica or David Kopf Instruments) and stainless steel injection cannulae (26 gauge, Plastics One) attached to syringes (catalog#80100, Hamilton) through polyethylene tubing were targeted bilaterally (unilaterally for retrograde tracer) to dlPAG (AP: − 7.2, DV: − 5.4, ML: + − 0.8) or vlPAG (AP: − 7.5, DV: − 6.2, ML: + − 0.8). For rabies viral or retrograde tracer (alexa 647 conjugated cholera toxin subunit B (Ctb-647) Invitrogen) injections (0.3 ul for both virus and tracer) into three different thalamic nuclei for manipulating or labeling thalamic projecting dlPAG neurons, the coordinates were as follows (aPVT: AP: − 1.8, DV: − 5.2, ML: +  − 1.9 at 20 degree angle, pPVT: AP: − 3.2, DV: − 5.2, ML: +  − 1.9 at a 20 degree angle, CM: AP: − 2.6, DV: − 6.4, ML: + − 1.13 at 10 degree angle). Retrograde tracers (viral or Ctb) were delivered unilaterally into the aPVT. After a 2 min wait period, injections (0.07 ul/min rate) were made and controlled by an automated pump (PHD2000, Harvard Apparatus), followed by a 15 min post-injection wait period. After injections were completed, bilateral optical cannulae (DFC_200/245_5.5mm_DF1.6_FLT, Doric Lenses) were targeted to the dl/lPAG (AP: − 7.2, DV: − 4.9, ML: +  − 0.8) or vlPAG (AP: − 7.5, DV: − 5.4, ML: +  − 0.8) and affixed to the skull using stainless steel surgical screws and dental cement.

### Behavioral conditioning experiments

For all auditory fear conditioning in behavioral studies, animals were placed into a sound isolating chamber (Med Associates) and received pairings of an auditory conditioned stimulus (CS) and electric shock unconditioned stimulus (US) during the training period. The CS for all experiments was a series of 85db, 5-kHz tone pips (at 1 Hz with 250 ms on and 750 ms off) for 20 s, and the foot shock US (0.7 mA) was presented concurrent with the final pip and lasted for 1 s. Presentation of both CS and US were controlled by custom made software (MED-PC, Med Associates). For the optogenetic manipulation, we checked that the laser intensity was 15-20mW from tips of optical fiber before each experiment.

For optogenetic cell body inactivation of dl or vlPAG experiments, 3 weeks after virus injection (AAV5-CAG-ArchT-GFP or AAV5-CAG-GFP) and optical cannula implant, animals were trained with 3 CS-US (0.7 mA) pairings. For optogenetic inactivation of thalamus projecting dlPAG neurons, training occurred 4–7 days following rabies virus injection. In all ‘Overlap’ and ‘GFP’ groups  orange laser (589 nm, Shanghai Laser) was provided to the target PAG area from 400 ms prior to US onset until 50 ms after US offset (total illumination time = 1.45 s). In the ‘Offset’ group, laser was given 50–70 s (pseudorandomly) after each shock period. During a testing period 24 h after training, animals received 3 CS alone presentations with pseudorandom inter-trial intervals (2.5 min on average). Rats’ freezing behavior during CS period during the test was scored using automated scoring software (Video Freeze, Med Associates). For experiments in which we inactivated aPVT projecting dlPAG neurons during expression of previously acquired fear responses, animals received 3 CS-US pairings. They then received 4 CS retrieval trials 24 h after training. During two of the CSs, orange laser illumination began 400 ms before CS onset and ended 400 ms after CS offset (total of 20.8 s) covered CS period (namely, 20 s). The presentation order of laser-CS and CS trials were counter-balanced across animals. The freezing level to laser-CS and CS without laser trials were averaged for each animal.

### Histological verification

To verify transgene (ArchT-GFP, GFP) expression and location of optical fiber tips and cannulae in targeted brain areas, rats were overdosed (with a 25% chloral hydrate) and perfused and tissue sections were cut after each experiment as described previously (see previous description [22]). For vl/dlPAG manipulation experiments in Fig. 1, an experimenter blind as to animal and treatment group assessed whether transgene expression occurred specifically in these regions and whether the tip of the optical fibers were dorsal and proximal to the target area. For the manipulation of thalamic projecting dlPAG cells experiments, an experimenter blind as to animal and treatment group assessed whether thalamic injection cannulae tracks were centered in the target structure and whether there were retrogradely labeled neurons evident in the dlPAG. If these criteria were not met, animals were not included in the analysis. To quantify the retrograde rabies viral tracing (Fig. 2D), all animals from the ‘Overlap’ groups of all behavioral experiments were used for analysis and cells were counted from every 3rd section from − 6.8 to − 7.6 AP from Bregma.

## Supplementary Information

**Additional file 1: Figure S1.A** Locations of optical fiber tips to deliver laser into dlPAG (cyan dots) or vlPAG (orange dots). **B**-**D** Locations of optical fiber tips to deliver laser into dlPAG to manipulate aPVT (B), CM (C) and pPVT (D) projecting dlPAG neurons. Overlap = cyan dots, Offset = red dots, GFP = green dots.

**Additional file 2: Figure S2**: **A**-**C** Graphs showing correlations between the number of dlPAG cells retrogradely infected from aPVT (**A**), pPVT (**B**) and CM (**C**) and the amount of tone evoked freezing following learning. **D** Example of retrogradely labeled dlPAG neurons projecting to the aPVT. Projections are labeled with Ctb 647 (white). **E** aPVT projecting dlPAG neurons are glutamatergic. Blue = NeuN, Red = vGluT2, White = Ctb 647, overlay in upper left panel with triangles indicating triple labeled neurons **F**-**G** Axon collaterals labeled with GFP from aPVT projecting dlPAG neurons in the dorsomedial hypothalamus (DMH), cuneiform nucleus (CnF) and parabrachial nucleus (PB). scp = superior cerebellar peduncle, 3 V = 3rd ventricle.

## Data Availability

Data will be made available upon reasonable request.
