# Supplementary material for: Functional organization of the midbrain periaqueductal gray for regulating aversive memory formation
Source: Mol Brain. 2021 Sep 8;14:136. doi: 10.1186/s13041-021-00844-0 (PMC8424891; doi:10.1186/s13041-021-00844-0)
Supplement: Supplementary file 1 — Additional file 1: Figure S1.A Locations of optical fiber tips to deliver laser into dlPAG (cyan dots) or vlPAG (orange dots). B-D Locations of optical fiber tips to deliver laser into dlPAG to manipulate aPVT (B), CM (C) and pPVT (D) projecting dlPAG neurons. Overlap = cyan dots, Offset = red dots, GFP = green dots. [file 13041_2021_844_MOESM1_ESM.docx]

**Additional File 1**


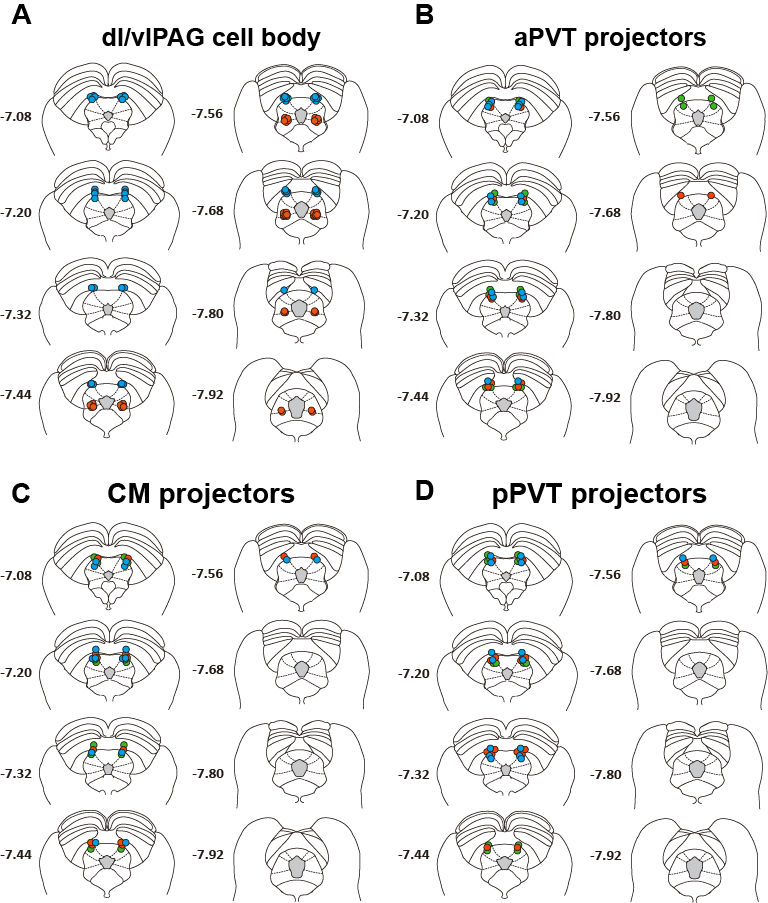


**Figure S1**: ***A***, Locations of optical fiber tips to deliver laser into dlPAG (cyan dots) or vlPAG (orange dots). Locations of optical fiber tips to deliver laser into dlPAG to manipulate aPVT (B), CM (C) and pPVT (D) projecting dlPAG neurons. Overlap=cyan dots, Offset=red dots, GFP=green dots.
