# Supplementary material for: Functional organization of the midbrain periaqueductal gray for regulating aversive memory formation
Source: Mol Brain. 2021 Sep 8;14:136. doi: 10.1186/s13041-021-00844-0 (PMC8424891; doi:10.1186/s13041-021-00844-0)
Supplement: Supplementary file 2 — Additional file 2: Figure S2: A-C Graphs showing correlations between the number of dlPAG cells retrogradely infected from aPVT (A), pPVT (B) and CM (C) and the amount of tone evoked freezing following learning. D Example of retrogradely labeled dlPAG neurons projecting to the aPVT. Projections are labeled with Ctb 647 (white). E aPVT projecting dlPAG neurons are glutamatergic. Blue = NeuN, Red = vGluT2, White = Ctb 647, overlay in upper left panel with triangles indicating triple labeled neurons F-G Axon collaterals labeled with GFP from aPVT projecting dlPAG neurons in the dorsomedial hypothalamus (DMH), cuneiform nucleus (CnF) and parabrachial nucleus (PB). scp = superior cerebellar peduncle, 3 V = 3rd ventricle. [file 13041_2021_844_MOESM2_ESM.docx]

**Additional File 2**


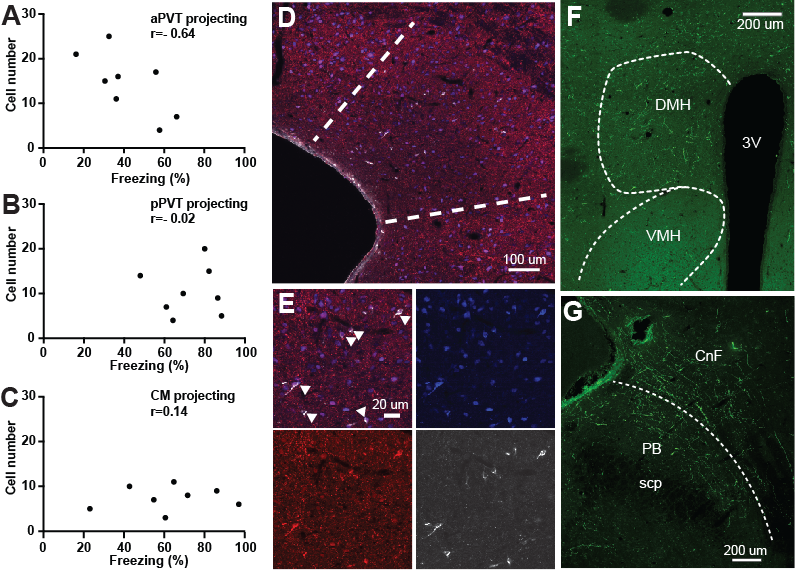


**Figure S2**: ***A-C***, Graphs showing correlations between the number of dlPAG cells retrogradely infected from aPVT (***A***), pPVT (***B***) and CM (***C***) and the amount of tone evoked freezing following learning. ***D***, Example of retrogradely labeled dlPAG neurons projecting to the aPVT. Projections are labeled with Ctb 647 (white). ***E***, aPVT projecting dlPAG neurons are glutamatergic. Blue=NeuN, Red=vGluT2, White=Ctb 647, overlay in upper left panel with triangles indicating triple labeled neurons ***F-G***, Axon collaterals labeled with GFP from aPVT projecting dlPAG neurons in the dorsomedial hypothalamus (DMH), cuneiform nucleus (CnF) and parabrachial nucleus (PB). scp=superior cerebellar peduncle, 3V=3^rd^ ventricle.
